# Fellows’ Perspective of Educational Changes in Medical Sports Medicine During the COVID-19 Pandemic

**DOI:** 10.7759/cureus.26199

**Published:** 2022-06-22

**Authors:** Cynthia J Stein, Ryan I Buller, Emily J Kivlehan, David N Williams, Mary E Dubon, Jill S Moschelli

**Affiliations:** 1 Division of Sports Medicine, Department of Orthopedics, Boston Children’s Hospital, Boston, USA; 2 Department of Orthopedics, Harvard Medical School, Boston, USA; 3 Family Medicine, University of California Riverside, Riverside, USA; 4 Physical Medicine and Rehabilitation, Spaulding Rehabilitation Hospital, Harvard Medical School, Boston, USA; 5 Institutional Centers for Clinical and Translational Research, Boston Children’s Hospital, Boston, USA; 6 Department of Biostatistics, Harvard Medical School, Boston, USA; 7 Division of Sports Medicine, Department of Orthopedics, Michigan State University, Okemos, USA

**Keywords:** sports, curriculum planning, qualitative and quantitative research, covid-19 pandemic, general well being, procedure training, graduate medical education (gme), online medical education, medical education, pediatric sports medicine

## Abstract

Background

As a result of the coronavirus disease 2019 (COVID-19) pandemic, graduate medical education, along with most of daily life, was disrupted. The goal of this study was to explore the experiences of fellows in primary care/medical sports medicine (MSM) and view the changes made to training programs through their eyes.

Methodology

A questionnaire was developed to collect qualitative and quantitative data regarding the fellow’s experiences in training from March to June 2020. Fellows on the American Medical Society for Sports Medicine list of current Sports Medicine Fellows in the United States and Canada were invited to participate. Of the 329 invited, 90 (27.4%) fellows returned questionnaires.

Results

MSM fellows highlighted positive adaptations as well as losses to their educational programs related to the pandemic. The biggest gain reported was additional learning time, and the greatest loss was to sports and event coverage. Most fellows reported attending collaborative sessions, and they noted an increase in didactics compared to pre-pandemic levels. The largest losses were noted in event coverage and training room exposures, with smaller declines in ultrasounds, procedures, and research experiences. They also described challenges, including changing clinical roles, managing social isolation and boredom, and balancing work and family responsibilities.

Conclusions

Fellows identified gains, losses, and challenges due to pandemic-related changes to their fellowship programs. Fellowship directors and educators in different fields can use this understanding of the fellows’ experiences to build on current resources, further develop collaborative efforts, create new educational opportunities, and provide additional support for fellow learning.

## Introduction

As the coronavirus disease 2019 (COVID-19) pandemic spread across North America in the late winter of 2020, graduate medical education (GME) programming, along with the rest of daily life, was disrupted. Social distancing, event cancellations, clinical reassignments, patient care adaptations, and quarantine orders created extraordinary circumstances and challenges.

Facing these abrupt and drastic changes, program directors looked for ways to support their trainees. They mobilized available resources and created new opportunities. Primary care/medical sports medicine (MSM) fellowships are particularly dependent on in-person interactions because fellowship is built around knowledge acquisition, expansion of clinical acumen, procedural skill development, and athletic event coverage, typically over a one-year period. Each element of fellowship presents its own unique challenges.

Multiple studies have been published on collaborative efforts and online resources during the pandemic [1-4]. One such study of pediatric rehabilitation medicine trainees found that the benefits of a national program of online learning included access to experts, networking, recording of lectures, and flexibility in terms of location. Concerns included a lack of protected time, online fatigue, and decreased levels of engagement [5].

Looking specifically at sports medicine, three studies of orthopedic sports fellowships demonstrated the effects of the dramatic drop in the number of surgical cases along with other pandemic-related changes [6-8]. One study examined the effects of the pandemic on the application process for orthopedic sports medicine fellowships [9]. Another study focused on the use of distance learning for ultrasound training during sports fellowship [10]. However, to our knowledge, the overall impact of the pandemic on MSM training has not been evaluated.

This study was designed to understand the MSM fellows’ experiences and develop a picture of sports medicine program modifications from the perspective of fellows. Additional objectives were to draw upon quantitative and qualitative data to identify both the challenges and possibly unforeseen benefits experienced during this time and to assist program directors and other educators as they plan future programming.

## Materials and methods

A questionnaire was developed using both multiple-choice and open-response questions (Appendices). The study was reviewed by the Internal Review Board (IRB) of a large teaching hospital in the United States and was determined to be IRB exempt. Anonymous study data were collected and managed using the REDCap (Research Electronic Data Capture) system hosted at the hospital [11,12]. There was no patient or public involvement in the development, implementation, or evaluation of this study.

The questionnaire was sent out on June 29, 2020, to 329 fellows in the United States and Canada through the American Medical Society for Sports Medicine (AMSSM) list of current Sports Medicine Fellows. An additional email describing the study was sent out to 228 program directors and associate directors to ask them to encourage fellow participation. No additional reminders were sent.

The introduction of the questionnaire explained the research objective, emphasized the fact that participation was voluntary, and stated explicitly that response to the questionnaire indicated implied consent to participate in the research study.

Qualitative data, in the form of responses to open-ended questions, were collected for descriptive analysis. Inductive thematic analysis was used to identify patterns in the responses, using the six stages described by Braun and Clarke [13], with the goal of describing and identifying these patterns and the broader applications of the data. Data were reviewed and coded (phases 1 and 2). Themes were identified, reviewed, defined, and named (phases 3, 4, and 5) and described in detail (phase 6). Initially, a single author (CJS) created the coding dictionary and identified the themes. A second author (DNW) coded the data and suggested modifications to the dictionary. Differences were discussed and reconciled.

## Results

In total, 90 fellows participated in the survey, representing a 27.4% response rate. Descriptive data on respondents are detailed in Table 1.

**Table 1:** Demographic and descriptive data of study participants (n = 90).

	n	%
Location (n = 72)		
United States, Northeast	20	27.8%
United States, Southeast	18	25.0%
United States, Midwest	18	25.0%
United States, Pacific	10	13.9%
United States, Southwest	6	8.3%
United States, Mountain	0	0.0 %
Canada	0	0.0%
Program length (n = 72)		
1 year	70	97.2%
2 years	2	2.8%
Primary specialty (n = 72)		
Family Medicine	37	51.4%
Physical Medicine & Rehabilitation	16	22.2%
Pediatrics	10	13.9%
Internal Medicine	6	8.3%
Emergency Medicine	3	4.2%

Quantitative data

Most fellows (78.6%) did not have a change in their clinical role related to the COVID-19 pandemic. Overall, 76% of fellows participated in telemedicine visits; none reported having participated in telemedicine visits prior to the pandemic.

Regarding education, 83% of respondents attended collaborative sessions. On average, fellows reported spending 2.8 hours/day involved in online learning (range: 0-8 hours/day). They indicated that the ideal number of hours/day (mean) was 1.9 (range: 0-5 hours/day).

The benefits of online education were ranked as follows: (1) Access to different educational opportunities (63%). (2) Decreased travel time (49%). (3) Recording of lectures (45%). (4) Access to experts (44%). (5) Efficiency (36%). (6) Networking opportunities (31%). (7) Other (1%).

They also ranked the biggest challenges of online education as follows: (1) Online fatigue (too many hours of online activity) (57%). (2) Missed procedures (57%). (3) Decreased interaction between faculty and trainees (37%). (4) Less engagement (33%). (5) Technological difficulties (32%). (6) Over scheduling (31%). (7) Decreased interaction among trainees (27%). (8) Other (1%).

The fellows were asked to quantify learning opportunities as abundant, adequate, minimal, or absent, both before and during the COVID-19 disruption (Table 2).

**Table 2:** Quantity of learning opportunities as categorized by fellows before (pre) and during (post) the COVID-19 disruption from March through June 2020 (n = 66). *: total responses = 65; **: total responses = 64. COVID-19: coronavirus disease 2019

	Abundant, n (%)	Adequate, n (%)	Minimal, n (%)	Absent, n (%)	N/A, n (%)
Clinical					
Pre	61 (92.4%)	5 (7.6%)	0	0	0
Post	11 (16.7%)	39 (59.1%)	13 (19.7%)	3 (4.5%)	0
Sports coverage					
Pre	54 (81.8%)	12 (18.2%)	0	0	0
Post*	1 (1.5%)	4 (6.2%)	10 (15.4%)	50 (76.9%)	0
Training room					
Pre	41 (62.1%)	20 (30.3%)	4 (6.1%)	1 (1.5%)	0
Post	1 (1.5%)	5 (7.6%)	13 (19.7%)	47 (71.2%)	0
Didactics					
Pre	35 (53.0%)	27 (40.9%)	4 (6.1%)	0	0
Post	41 (62.1%)	23 (34.8%)	2 (3.0%)	0	0
Ultrasound					
Pre	36 (54.5%)	26 (39.4%)	4 (6.1%)	0	0
Post	24 (36.4%)	28 (42.4%)	13 (19.7%)	1 (1.5%)	0
Procedures					
Pre	41 (62.1%)	23 (34.8%)	2 (3.0%)	0	0
Post	17 (25.8%)	29 (43.9%)	13 (19.7%)	7 (10.6%)	0
Research					
Pre*	16 (24.6%)	35 (53.8%)	12 (18.4%)	0	2 (3.1%)
Post**	22 (34.3%)	24 (37.5%)	15 (23.4%)	3 (4.5%)	0

Fellows rated their satisfaction with their training programs as follows: very satisfied (34%), satisfied (37%), neutral (16%), dissatisfied (11%), and very dissatisfied (2%). They rated the perceptions of their fellowship’s attention to fellow education as follows: excellent (61%), good (27%), fair (11%), poor (0%), and very poor (1%).

Overall, 77% (51/66) of the fellows indicated that they did not have concerns about their skills or experience related to the pandemic disruption, and none reported having their training programs extended due to the pandemic.

Qualitative data

In total, 64 people responded to the open-ended question “What do you feel was the biggest loss to your education as a result of the pandemic?.” After coding the individual answers, four main themes were identified. Sports/Event coverage was the most common answer, mentioned by 30 of 64 fellows, which included “spring sports,” “training room experience,” and “sideline coverage.” Three additional answer topics included procedural learning (28/64) “refining my procedural skills,” “experience with ultrasound-guided injections”; decreased patient contact (21/64) “clinic volume,” “face-to-face patient encounters,” and “seeing patients and more pathology”; and loss of meetings and conferences (8/64) “loss of conference experience and poster presentations,” and “inability to attend the annual meetings, present in person.”

Overall, 62 people responded to the open-ended question: “What do you feel was the biggest improvement to your education that resulted from the pandemic-related changes?.” Three main themes emerged. The most common response was more time for studying and self-directed learning submitted by 34 fellows: “increased time for reading, board exam preparation, and utilization of … online resources.” “I read way more journal articles during the pandemic than I would have otherwise.” “More time for ultrasound training,” and “Increased time for research.” The second most common response (12/64) was an increased amount and variety of learning opportunities: “Attending national lectures from experts in the field.” “Collaboration with other programs for education.” “Didactics provided me more learning on things I don’t normally cover day-to-day.” “New virtual radiology lectures and collaborative journal clubs.” The third theme (9/64) centered on opportunities using telemedicine: “Trial period with telehealth,” and “More comfort with performing virtual visits.”

In total, 53 fellows responded to the open-ended question: “What was your biggest personal or professional challenge during this time?.” The data highlighted several themes. Most commonly mentioned (25/53) were challenges related to training and clinical roles, which took several forms: “Juggling the addition of online training and clinical duties. I didn’t want to miss learning opportunities but I was in the clinic and doing online training, sometimes at the same time.” “Having to develop a skilled telemed exam.” “Feeling incompetent due to loss of clinical experience.” “Loss of procedural training … now I worry about beginning my career in sports medicine as an attending with comfort in only ‘basic’ procedures.”

The second most common response (8/53) was related to boredom and social isolation: “lack of social interaction,” “adjusting to isolation,” “too much downtime is boring,” “really just missed the in-person interaction with colleagues, patients…,” and “…lack of sports and other events that were engaging and entertaining.”

Childcare and family responsibilities were the third most common response (7/53): “very difficult without childcare (due to daycare closures),” “managing full-time childcare and trying to be present for online educational activities,” and “childcare and working from home.”

Additional concerns were also mentioned: COVID-19 risk/exposure (5/53), job search (4/53), and general uncertainty (3/53).

## Discussion

This study demonstrated the overall positive perceptions of MSM fellow education during the early stages of the COVID-19 pandemic, highlighting the adaptability of MSM training programs and the resiliency of MSM fellows. During this period, MSM programs shifted largely to online activities and self-study. Most fellows indicated that they were satisfied with their education and did not express concerns about their skills or experience related to the pandemic disruption.

While generally satisfied, survey responses also provided a picture of significant challenges that the fellows faced. The use of open-ended questions allowed participants to respond using their own words without being limited to or influenced by specific answer choices. The qualitative data complemented the quantitative findings. Evident in fellow responses regarding losses during the disruption were two main concepts: (1) Missed practical experiences, and (2) lost opportunities for enjoyment. Following the restrictions on in-person contact and the cancellation of most public events, the fellows described limited patient contact, reduced ultrasound and procedural practice, lost opportunities to work with athletes on the field and in the training room, inability to attend sporting events, and missed chances to participate in national conferences.

**Figure 1:**
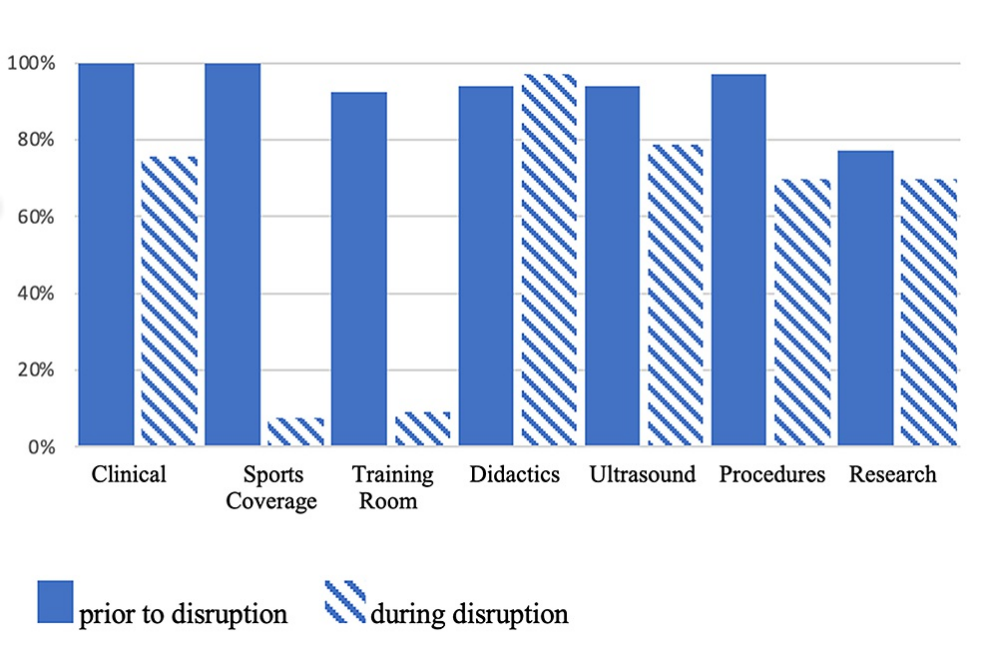
Bar graph representing the percentage of answers indicating “abundant” or “adequate” opportunities in different areas of training prior to and during the COVID-19 disruption. COVID-19: coronavirus disease 2019

These marked changes in learning opportunities are demonstrated in Figure 1. Overall, 100% of respondents indicated that prior to the pandemic they had abundant or adequate clinical exposure and sports coverage, and over 90% reported abundant or adequate opportunities in the training room, didactics, ultrasound, and procedures. Research was the only category with a lower percentage of abundant or adequate opportunities. Not surprisingly, following the COVID-19 restrictions, the largest drops (from abundant/adequate to minimal/absent) were in sports coverage (100% to 7.7%) and training room coverage (92.4% to 9.1%). The only area of increase was didactics from 93.9% abundant/adequate pre-pandemic to 97% during the pandemic.

This increase in didactic opportunities was one of several positive outcomes also noted in the qualitative data. The pandemic disruption freed many trainees from their typical scheduling constraints and patient care responsibilities. Two overarching themes emerged from the question about the largest educational improvement: (1) Increased study time: new-found time and flexibility allowed for self-directed study (research, board review, ultrasound practice), and (2) New opportunities in terms of venues and topic selections. The disruption of regular programming brought opportunities such as virtual sessions, online resources, journal clubs, and local and national collaborations. Increased accessibility to teaching from national experts was highlighted, also noted by trainees in other specialties [5]. Additional positive changes mentioned were experiences with telemedicine and time to spend with patients.

Over 80% of respondents participated in collaborative programs. Although the survey did not ask specifically if fellows had been involved in collaborative programs prior to the pandemic, the authors are aware that programs were developed and/or made more widely available in response to the COVID-19 pandemic disruption. These included virtual lectures and journal clubs that were offered online to programs nationally.

Fellows reported both positive and negative impacts of online activities. The main benefit was access to different educational opportunities, followed by decreased travel time, recording of lectures, and access to experts. As in-person patient care and education return, program directors may want to build on these collaborative efforts and online resources.

Future online curricula should also consider those elements that were most taxing to trainees, especially online fatigue. Fellows reported an average of 2.8 hours of online learning per day, but they indicated that the ideal would be 1.9 hours/day. This information, in conjunction with other commonly reported negative aspects of online education, decreased interaction between faculty and trainees, less engagement, technological difficulties, overscheduling, and decreased interaction among trainees, should be considered in future scheduling. In addition, programs should make every effort to avoid merely transferring in-person lectures to online presentations but focus on improving the delivery of material to encourage information processing and application.

The importance of active learning has been well documented [14], along with well-described elements and benefits of electronic learning [15]. Many published recommendations and guidelines are available to educators [16-18]. Practical techniques have been described both for in-person didactics [19] and online/technology-enhanced education [20-22]. For example, increased self-directing learning, followed by flipped classroom sessions, has been received positively by medical students during virtual clinical rotations [23].

Because of the pandemic, the classic models of care delivery have been expanded. One clear example is the prevalence of telemedicine experiences. The majority of fellows (76.1%) participated in telemedicine visits, while none reported having been involved in telemedicine prior to the pandemic.

Another area of significant interest is in increasing options for procedural education [24]. The reduced number of procedural opportunities described by MSM fellows aligns with findings in other procedure-rich specialties [25]. Given the need to limit in-person contact, it was not surprising that loss of procedures was one of the most commonly reported limitations in both the qualitative and quantitative data. This deficit may be most easily addressed as fellows are able to return to in-person patient care. However, developing online, simulation, and other technology-enhanced methods can greatly support in-person education and help standardize trainee experiences.

Fellowship programs should remain cognizant of the stressors affecting fellows, especially in times of rapid and unwelcome change. This study highlighted a variety of personal and professional challenges. Three overarching themes emerged: (1) Scheduling challenges. Fellows describe being both under- and over-scheduled. They describe boredom, monotony, concerns about getting adequate training given the loss of clinical and procedural time, difficulty using time efficiently, and competing demands on their time. They present a picture of juggling childcare and other family responsibilities with online education, in-person clinical requirements, and new or changing telehealth demands. (2) Uncertainty. Fellows faced many different sources of uncertainty. From a perceived lack of educational guidance, changes in training schedules with decreased clinic visits and procedures, and altered and ambiguous clinical roles to concern about COVID-19 risk for themselves and their families. They also indicated concern over job search and preparedness for independent practice. (3) Isolation. The isolating effects of the pandemic were also evident in the fellows’ responses. Several described living and working alone, missing interactions with patients and colleagues, and feeling the loss of social interactions.

Fellowship programs should strive to prevent or address these stressors directly. There is growing literature on the training experience and recommendations for optimizing education and wellbeing [5,26]. Based on this study’s findings, it is important to avoid overscheduling, overlapping commitments, and long periods without educational activities. Where possible, flexibility may allow fellows to better balance program requirements with family and other external commitments. Combining synchronous and asynchronous learning can provide some of this flexibility.

Increasingly, there are published reports of anxiety, stress [27,28], depression, and burnout [25] among healthcare trainees during the pandemic. Fellows in this study identified multiple causes of stress and isolation. Programs may be able to address modifiable factors, maintain regular communication, provide updates as needed, and facilitate channels to discuss new concerns as they arise. Group activities and one-on-one sessions for communication, problem-solving, and interpersonal interaction may also increase engagement and reduce feelings of isolation.

Study limitations

This study has several limitations. The questionnaire was sent in June 2020 at the end of most fellowship programs. Fellows received a single email with no reminders, which likely reduced the number of responses. This lower response rate affected generalizability. However, the information gathered, especially in the form of the qualitative data, highlights several important themes and allows for deeper understanding of fellows’ experiences.

The timing of the pandemic and the questionnaire likely affected the study results in other ways as well. Only current MSM fellows were eligible to participate in the study. Almost all were in one-year programs and had completed eight to nine months of fellowship prior to the pandemic disruption. Therefore, fellows were able to build on the foundation of the first part of their training and may have been better able to adjust to the changes. This may have affected their satisfaction scores and other assessments of their training.

Another limitation of this study was the lack of demographic details collected from respondents. Future studies would benefit from detailed demographic data, especially age and gender, that likely impact preference and access to training opportunities, types of stressors and supports, and levels of satisfaction with educational programming.

## Conclusions

This study provides a view of MSM fellows’ lives and training during the period of rapid change from March to June 2020. As expected, fellows experienced a variety of educational limitations, especially in terms of event coverage, training room time, and procedural skills practice. They also described isolation, boredom, and uncertainty along with the struggle to balance work, education, and family responsibilities. In addition, fellows highlighted positive changes, which included increased didactics and time for learning, along with access to collaborative sessions and remote experts. Program directors and other educators can use this information to better recognize opportunities and challenges faced by fellows and to further develop educational options. Even as in-person education and coverage return, asynchronous, virtual, and collaborative elements can help support and enrich medical training. Additional work is needed, especially to standardize and improve the teaching of manual and procedural skills and to reduce and prevent isolation, anxiety, and burnout among trainees.

## Appendices

**Table 3:** Questionnaire emailed to medical sports medicine fellows.

This survey is designed to gather your feedback on primary care sports medicine fellowship education during the ongoing COVID-19 pandemic (from March 2020 until the present date). The survey should take less than 4 minutes to complete. By completing this survey, you are agreeing to participate in a research study. Participation is voluntary and separate from your fellowship program. Your decision on whether or not to participate will have no effect on your standing in your program. This survey is anonymous and no personally identifiable information will be collected. If you have any questions, please feel free to contact Dr. Cynthia Stein, MD, MPH at .	
Question	Response choices
1. I understand and agree to participate in this research project.	a. Yes
	b. No
2. What year are you in fellowship?	a. 1
	b. 2
3. What is the length of your fellowship program?	a. 1 year
	b. 2 years
4. How many primary care sports medicine fellows are in your fellowship program?	a. 1
	b. 2
	c. 3
	d. 4
	e. 5 or more
5. Where is your fellowship based?	a. Northeast US (ME, NH, VT, MA, RI, CT, NY, NJ, PA)
	b. Southeast US (DE, MD, VA, WV, KY, NC, SC, TN, MS, AL, GA, FL, AR, LA)
	c. Midwest US (MI, OH, IN, WI, IL, MN, IA, MO, ND, SD, NE, KS)
	d. Southwest US (OK, TX, NM, AZ)
	e. Mountain US (MT, WY, CO, ID, UT, NV)
	f. Pacific US (WA, OR, CA, AK, HI)
	g. Canada
6. What is your primary specialty? (What type of residency program did you complete?)	a. Family Medicine
	b. Pediatrics
	c. Internal Medicine
	d. Emergency Medicine
	e. Physical Medicine and Rehabilitation
	f. Other
6b. (If other) Please specify:	Open Response
7. Were you moved into a different role to cover patient care needs during this time?	a. Yes
	b. No
7b. (If yes) What was your new role?	Open Response
8. Throughout this period of the pandemic, have you continued to see patients in person in sports medicine clinics?	a. Yes
	b. No
8b. (If yes) On average, how many patients have you seen in person?	a. Fewer than before the pandemic
	b. About the same number as before the pandemic
	c. More than before the pandemic
9. Did you see patients via telemedicine visits as a result of COVID-19?	a. No
	b. Yes, I started seeing patients via telemedicine as a result of COVID-19
	c. Yes, I was seeing patients via telemedicine before the pandemic and have continued
9a. (If yes, started as a result of the pandemic) Approximately how many patient virtual visits have you had per week?	Open Response
9b. (If yes, seeing patients via telemedicine before and during the pandemic) Approximately how many patient virtual visits did you have per week before the pandemic?	Open Response
9c. (If yes, seeing patients via telemedicine before and during the pandemic) Approximately how many patient virtual visits did you have per week during the pandemic?	Open Response
10. Has your fellowship been extended because of the pandemic?	a. Yes
	b. No
10b. (If yes) How much has it been extended by?	Open Response
10c. (If yes) Select unit	a. Days
	b. Months
	c. Years
11. BEFORE any pandemic-related changes were made to your fellowship program, how would you rate each of the following elements of your training?	
Clinical experience	a. Abundant
	b. Adequate
	c. Minimal
	d. Absent
	e. N/A
Sports/event coverage	a. Abundant
	b. Adequate
	c. Minimal
	d. Absent
	e. N/A
Athletic training room experience	a. Abundant
	b. Adequate
	c. Minimal
	d. Absent
	e. N/A
Didactic education	a. Abundant
	b. Adequate
	c. Minimal
	d. Absent
	e. N/A
Ultrasound experience	a. Abundant
	b. Adequate
	c. Minimal
	d. Absent
	e. N/A
Procedural experience	a. Abundant
	b. Adequate
	c. Minimal
	d. Absent
	e. N/A
Research	a. Abundant
	b. Adequate
	c. Minimal
	d. Absent
	e. N/A
12. AFTER any pandemic-related changes were made to your fellowship program, how would you rate each of the following elements of your training?	
Clinical experience	a. Abundant
	b. Adequate
	c. Minimal
	d. Absent
	e. N/A
Sports/event coverage	a. Abundant
	b. Adequate
	c. Minimal
	d. Absent
	e. N/A
Athletic training room experience	a. Abundant
	b. Adequate
	c. Minimal
	d. Absent
	e. N/A
Didactic education	a. Abundant
	b. Adequate
	c. Minimal
	d. Absent
	e. N/A
Ultrasound experience	a. Abundant
	b. Adequate
	c. Minimal
	d. Absent
	e. N/A
Procedural experience	a. Abundant
	b. Adequate
	c. Minimal
	d. Absent
	e. N/A
Research	a. Abundant
	b. Adequate
	c. Minimal
	d. Absent
	e. N/A
13. Did you participate in new collaborative, regional, or national educational programs (journal clubs/lectures/conferences) as a result of the pandemic?	a. Yes
	b. No
14. (If yes) Which programs did you participate in?	Open Response
15. What do you feel was the biggest loss to your education as a result of the pandemic?	Open Response
16. What do you feel was the biggest improvement to your education that resulted from the pandemic-related changes?	Open Response
17. Compared to pre-pandemic, how satisfied are you with the quality of your education during the pandemic?	a. Very satisfied
	b. Satisfied
	c. Neutral
	d. Dissatisfied
	e. Very dissatisfied
18. How would you rate the attention paid to your educational needs by your fellowship program during this time?	a. Excellent
	b. Good
	c. Fair
	d. Poor
	e. Very poor
19. On average, how many hours per day were you involved in online learning?	a. 0
	b. 1
	c. 2
	d. 3
	e. 4
	f. 5
	g. 6
	h. 7
	i. 8
	j. 9 or more
20. What do you think would be the ideal number of hours of online education per day?	a. 0
	b. 1
	c. 2
	d. 3
	e. 4
	f. 5
	g. 6
	h. 7
	i. 8
	j. Other
20b. (If other) Please specify:	Open Response
21. For you, what are the benefits of online fellowship education? (Check all that apply.)	a. Efficiency
	b. Access to different educational opportunities
	c. Access to experts
	d. Decreased travel time
	e. Networking opportunities
	f. Recording of lectures
	g. Other
21b. (If other) Please specify:	Open Response
22. For you, what are some of the challenges of online fellowship education? (Check all that apply.)	a. Technological difficulties
	b. On-line fatigue (too many hours of online activity)
	c. Over scheduling
	d. Less engaging
	e. Decreased interaction among trainees
	f. Decreased interaction between faculty and trainees
	g. Missing procedures
	h. Other
22b. (If other) Please specify:	Open Response
23. What was your biggest personal or professional challenge during this time?	Open Response
24. Do you have any concerns about your skills/experience that resulted from the pandemic disruption?	a. Yes
	b. No
24b. (If yes) What are your concerns about your skills/experience?	Open Response
